# *Ganoderma lucidum* Sporoderm-Broken Spore Powder (GLSP) Remodels the Gut Microbiota and Promotes Tryptophan-Derived Indole Metabolites to Maintain Intestinal Homeostasis

**DOI:** 10.3390/microorganisms14102280

**Published:** 2026-10-07

**Authors:** Miao Zheng, Rui Chen, Yuannan Wei, Yufeng Zeng, Dan Wang, Yuanyuan Ren, Xinting Wang, Ling Liu, Xi Yang, Lei Shi, Lingbiao Xin, Jie Yang

**Affiliations:** 1 State Key Laboratory of Experimental Hematology, Key Laboratory of Cellular and Molecular Immunology in Tianjin, and Key Laboratory of Immune Microenvironment and Disease (Ministry of Education), Key Laboratory of Breast Cancer Prevention and Therapy (Ministry of Education), The Province and Ministry Co-Sponsored Collaborative Innovation Center for Medical Epigenetics, Tianjin Medical University, Tianjin 300071, China; 2 Department of Immunology, School of Basic Medical Science, Tianjin Medical University, Tianjin 300071, China; 3 Department of Immunology, University of Manitoba, Winnipeg, MB R3E 0T5, Canada; 4 Department of Biochemistry and Molecular Biology, School of Basic Medical Science, Tianjin Medical University, Tianjin 301700, China

**Keywords:** *Ganoderma lucidum* spore powder, intestinal barrier, gut microbiota, tryptophan metabolism, indole

## Abstract

*Ganoderma lucidum* sporoderm-broken spore powder (GLSP) has been historically documented for its anti-aging and anti-tumor effects. Although several studies have reported that GLSP and related substance may improve physiological function by regulating the gut microbiota, the underlying mechanisms and their effect on intestinal function remains largely unknown. In this study, we comprehensively investigated the impact of dietary GLSP on intestinal epithelial dynamics and barrier function, assessed by single-cell RNA sequencing (scRNA-seq), using murine models spanning physiological homeostasis and DSS-induced intestinal injury. Our study reveals that GLSP protects intestinal epithelia against chemical and age-related barrier injury by promoting the coordinated expansion of stem/progenitor compartments and restoring both the mucus and physical barriers. Gut microbiota involvement was confirmed via antibiotic-treated mice and fecal microbiota transplantation (FMT). FMT from GLSP-diet donors successfully recapitulated the protective effects in gut microbiota-depleted recipient mice. Multi-omics integration of 16S rRNA sequencing and metabolomic profiling identified the enrichment of SCFA-producing genera, and previously unrecognized microbial metabolites, such as indole-2-carboxylic acid, 3-Methyloxindole, and 4-hydroxyindole. These microbial metabolites are likely to play an essential role in reprogramming the intestinal metabolome, particularly tryptophan–indole metabolic pathways, to mediate intestinal homeostasis. These findings provide novel insights and potential directions for the development of new drugs targeting intestinal injury.

## 1. Introduction

The intestinal epithelium serves as a critical physical and biochemical barrier that separates the host from the luminal environment, playing an indispensable role in nutrient absorption, immune surveillance, and defense against pathogenic invasion [1,2]. This barrier is maintained by a coordinated interplay among diverse epithelial cell types, including intestinal stem cells (ISCs) at the crypt base, transit-amplifying (TA) progenitor cells, and terminally differentiated secretory cells such as goblet cells and Paneth cells. ISCs, marked by LGR5, continuously self-renew and generate progeny that differentiate into specialized lineages, ensuring the rapid epithelial turnover required for barrier integrity [3,4]. Goblet cells secrete mucins, predominantly MUC2, which form a mucus layer that shields the intestinal epithelium from microbial encroachment and harmful chemical agents [5,6].

The gut microbiota, a complex ecosystem of trillions of microorganisms inhabiting the gastrointestinal tract, is essential for maintaining intestinal homeostasis [7,8]. In addition to directly supporting the intestinal barrier by competing for ecological niches, commensal bacteria produce a vast array of metabolites, including short-chain fatty acids (SCFAs), bile acid derivatives, and tryptophan catabolites, which modulate epithelial proliferation, differentiation, and immune responses [9]. Among these, tryptophan-derived indole metabolites have garnered particular attention for their potential aryl hydrocarbon receptor (AhR)-mediated enhancement of barrier function and IL-22-driven mucosal repair [10]. Consequently, strategies that harness the microbiota–metabolite–epithelium axis offer compelling therapeutic potential for intestinal barrier disorders.

Natural products and their active ingredients have attracted widespread attention owing to their favorable biocompatibility, diverse bioactivities, and multi-target regulatory properties [11,12,13]. *Ganoderma lucidum* (*Lingzhi*), a renowned medicinal mushroom that has been used in traditional Asian medicine for millennia, has been extensively studied for its diverse pharmacological activities, including immunomodulation, anti-tumor, hepatoprotective, and anti-inflammatory effects [14]. The *Ganoderma lucidum* sporoderm-broken spore powder (GLSP) obtained from the mature basidiospores of the mushroom is particularly rich in bioactive polysaccharides, triterpenoids, proteins, and amino acids [15,16]. Recent investigations have begun to uncover the potential of *Ganoderma lucidum* spore polysaccharides in modulating intestinal health. Studies have shown that it can ameliorate obesity-induced intestinal barrier impairment by reducing serum lipopolysaccharide (LPS) levels, increasing the expression of tight junction proteins such as Claudin-1 and ZO-1, and enriching probiotics [17]. In addition, Oil-extracted GLSP has been reported to alleviate microbiota dysbiosis by enriching *Bacteroidetes*, *Firmicutes*, *Lactobacillus*, and *Roseburia*, thus improving intestinal barrier function [18]. Notably, BGLSP-B2, an active fraction purified from sporoderm-broken *Ganoderma lucidum* spore polysaccharides, has been identified as a key component that alleviates DSS-induced colitis in mice by restoring intestinal barrier integrity, modulating gut microbiota, and regulating Th17/Treg homeostasis [19]. Despite these advances, the comprehensive mechanisms by which GLSP, particularly through its effects on the gut microbiota and its metabolites, contributes to intestinal homeostasis remain incompletely understood.

Therefore, the present study aimed to systematically elucidate the mechanisms through which dietary GLSP maintains intestinal homeostasis and protects the epithelial barrier. We hypothesized that GLSP exerts its protective effects by remodeling the gut microbiota and reprogramming microbiota-derived metabolism—particularly the tryptophan–indole pathway—thereby reinforcing both the mucus and physical barriers and promoting epithelial repair. To test this, we comprehensively investigated the impact of a GLSP diet on intestinal epithelial dynamics and barrier function using murine models of physiological homeostasis and DSS-induced intestinal injury. By integrating single-cell transcriptomics, microbiota profiling, metabolomics, and fecal microbiota transplantation (FMT), we demonstrate that the protective effects of the GLSP diet are dependent on the gut microbiota and are specifically linked to the enrichment of SCFA-producing genera and the concomitant elevation of indole metabolites. These findings not only elucidate a novel mechanism for GLSP action but also identify indole-2-carboxylic acid, 3-Methyloxindole, and 4-hydroxyindole as previously unrecognized microbial metabolites that likely contribute to intestinal protection.

## 2. Materials and Methods

### 2.1. Mice

Male C57BL/6J mice (3 weeks, 6 weeks, and 8 months old) were purchased from Beijing Vital River Laboratory Animal Technology Co., Ltd. (Beijing, China). The animals were housed under specific pathogen-free (SPF) conditions at Tianjin Medical University with a 12 h light/dark cycle. All procedures were approved by the Animal Care and Use Committee of Tianjin Medical University (TMUaMEC 2023024).

### 2.2. Model Establishment

For dietary intervention, the *Ganoderma lucidum* sporoderm-broken spore powder (GLSP) diet was prepared by uniformly mixing 4 g/kg (0.4%) of the spore powder into the basal control diet. This formulation was commissioned to Kuibu Qianli Biotechnology (Xuancheng) Co., Ltd. (Xuancheng, China). The CON diet was matched to the GLSP diet in energy, protein, fat, carbohydrate, and fiber composition, differing only by the absence of GLSP; the GLSP used was from a single batch under the brand name Jianyinian (China). In all experiments involving spore powder administration, mice were fed the supplemented diet starting 10 weeks prior to any subsequent procedures. To induce colitis, mice were given free access to drinking water containing dextran sulfate sodium (DSS; MP Biomedicals, Santa Ana, 0216011080, CA, USA) at concentrations of 1.5% (*w*/*v*) for chronic colitis or 2.5% (*w*/*v*) for acute colitis, as appropriate.

### 2.3. Assessment of Disease Activity Index (DAI)

DAI was used to assess disease severity by monitoring body weight loss, stool consistency, and fecal bleeding. During the dextran sulfate sodium (DSS)-induced modeling phase, the DAI score was recorded daily for each mouse. Body weight loss was scored as follows: <2% loss (0 points), 2–5% loss (1 point), 6–10% loss (2 points), 11–15% loss (3 points), >15% loss (4 points). Stool consistency was scored as follows: normal (0 points), soft stool (1 point), mucous stool (2 points), loose stool (3 points), watery diarrhea (4 points). Fecal bleeding was assessed based on the presence of blood in the stool: normal (0 points), occult blood positive (1 point), mild bleeding (2 points), obvious bleeding (3 points), gross blood at the anus (4 points). According to the above criteria, the DAI score was determined as the sum of the three subscores.

### 2.4. HE and Alcian Blue Staining

Colon tissue samples were fixed in 4% paraformaldehyde for 48 h, dehydrated, and embedded in paraffin. Paraffin-embedded tissues were sectioned at 4 μm, deparaffinized, and stained with H&E or Alcian blue staining (Beyotime, C0153S, Beijing, China). Images were acquired at 20×, and goblet cells were quantified from Alcian blue-stained sections using ImageJ (Fiji Is Just).

### 2.5. Histopathological Assessment

The histopathological score was used as the primary criterion for evaluating the degree of inflammation, and the score was as follows: Ulcer formation area was scored as follows: 0% (0 points), 0–5% (1 point), 5–10% (2 points), 10–20% (3 points), >20% (4 points). Epithelial cell changes were scored as follows: normal (0 points), goblet cell loss (1 point), extensive goblet cell loss (2 points), crypt loss (3 points), extensive crypt loss or polypoid regeneration (4 points). Inflammatory infiltration was scored as follows: none (0 points), pericryptal infiltration (1 point), infiltration of mucosal muscle layer (2 points), widespread infiltration of the muscularis mucosae with mucosal thickening (3 points), infiltration into the submucosa (4 points). According to the above criteria, the histopathological score was determined as the sum of the three subscores, with a maximum possible score of 12.

### 2.6. LPS Assay

Serum lipopolysaccharide (LPS) levels were quantified using enzyme-linked immunosorbent assay (ELISA) (CSB-E13066m, Cusabio, Wuhan, China). Briefly, after anesthesia, blood was collected from mice via orbital bleeding and allowed to stand for 4 h. Serum was then separated by centrifugation at 4 °C at 1500r for 20 min. A commercial ELISA kit was used according to the manufacturer’s instructions to determine LPS concentration in serum.

### 2.7. Total RNA Extraction and Real-Time Quantitative PCR

Total RNA was extracted from colon tissues using TRIzol reagent and reverse-transcribed into cDNA for qRT-PCR analysis. Real-time PCR was then performed using SYBR Green Supermix with specific primers for IL-6 [5′-TACCACTTCACAAGTCGGAGGC-3′ (Forward), 5′-CTGCAAGTGCATCATCGTTGTTC-3′ (Reverse)], IL-1β [5′-TGGACCTTCCAGGATGAGGACA-3′ (Forward), 5′-GTTCATCTCGGAGCCTGTAGTG-3′ (Reverse)], TNF-α [5′-GGTGCCTATGTCTCAGCCTCTT-3′ (Forward), 5′-GCCATAGAACTGATGAGAGGGAG-3′ (Reverse)] and β-Actin [5′-GTGACGTTGACATCCGTAAAGA-3′ (Forward), 5′-GCCGGACTCATCGTACTCC-3′ (Reverse)] on a LightCycler^®^ 96 Instrument.

### 2.8. Transmission Electron Microscopy (TEM) Analysis

Colon tissues were first fixed in 3% glutaraldehyde at 4 °C for 24–48 h, and subsequent sample processing was conducted by Fanrui Yunzhi Technology Co., Ltd. (Zhengzhou, China). Ultrastructural imaging was performed with a transmission electron microscope. Quantitative analysis of colonic microvilli and mitochondria was carried out using ImageJ software.

### 2.9. Immunofluorescence (IF) Staining

Deparaffinized sections were incubated overnight at 4 °C with primary antibodies against Claudin-1 (Proteintech, 28674-1-AP, Wuhan, China), Occludin (Proteintech, 27260-1-AP, Wuhan, China), ZO-1 (Proteintech, 21773-1-AP, Wuhan, China), E-cadherin (BD Biosciences, Franklin Lakes, 610182, NJ, USA), β-catenin (Cell Signaling Technology, 8480S, MA, USA), LGR5 (Proteintech, 30007-1-AP, Wuhan, China), and Ki-67 (Proteintech, 28074-1-AP, Wuhan, China). After washing, sections were incubated with fluorescence-labeled secondary antibodies (anti-rabbit Alexa Fluor™ 488, A21206; anti-mouse Alexa Fluor™ 546, A10036; Thermo Fisher Scientific, MA, USA) at 4 °C for 8 h, followed by DAPI (1:1000, Sigma-Aldrich, St. Louis, MO, USA) for 3 min. Sections were then mounted with anti-fade medium (Beyotime, P0131-5ml) and imaged under a fluorescence microscope (Leica DMi8, Wetzlar, Germany).

### 2.10. Single-Cell RNA Sequencing Analysis

After 10 weeks of GLSP feeding, colonic crypt tissues from three mice per group were pooled into a single sample for each of the CON and GLSP-diet groups. Viable single-cell suspensions were then prepared from the pooled colonic crypts and used for scRNA-seq library generation (10× Genomics Chromium platform). Thus, one pooled sample was obtained per group without biological replicates. The libraries were quality-checked and sequenced on an Illumina sequencing platform by Beijing Zhongkangbo Biotechnology Co., Ltd. (Beijing, China). according to the standard protocol outlined in the manual. The sequencing and basic data analysis were loaded into the R package Seurat version 2.3.4, which allows for the selection and filtration of cells on the basis of quality control metrics, data normalization and scaling, and detection of highly variable genes.

### 2.11. 16S rRNA Gene Sequencing

To investigate the effects of a GLSP diet on the gut microbiota of mice, fresh fecal samples were collected from the CON group (*n* = 6) and the GLSP-diet group (*n* = 6), placed in sterile cryotubes, and stored at −80 °C within 3 h of collection. Total genomic DNA was extracted using the TGuide S96 Magnetic Soil/Stool DNA Kit (Tiangen Biotech (Beijing, China) Co., Ltd.) according to the manufacturer’s instructions. DNA quality was assessed by 1.8% agarose gel electrophoresis, and DNA concentration and purity were determined using a NanoDrop 2000 UV-Vis spectrophotometer (Thermo Scientific, Wilmington, NC, USA). The V3-V4 hypervariable region of the bacterial 16S rRNA gene was targeted and amplified for subsequent analysis. The above steps and the subsequent sequencing analysis were performed by Beijing Zhongkangbo Biotechnology Co., Ltd.

### 2.12. Untargeted Metabolomics

To investigate the effects of a GLSP diet on the metabolic profile of mice, fresh fecal samples were collected from the CON group (*n* = 6) and the GLSP-diet group (*n* = 6), placed in sterile cryotubes, and stored at −80 °C within 3 h of collection. Metabolite extraction was performed using a Waters Acquity I-Class PLUS ultra-high performance liquid chromatography system coupled to a Waters Xevo G2-XS QTof high-resolution mass spectrometer (Waters, Milford, MA, USA), with a Waters Acquity UPLC HSS T3 column (Waters, MA, USA); the Waters Xevo G2-XS QTOF high-resolution mass spectrometer was controlled by the acquisition software (MassLynx V4.2, Waters, MA, USA) to collect primary and secondary mass spectrometry data in MSe mode for subsequent analysis. This untargeted metabolomics analysis was performed by Beijing Zhongkangbo Biotechnology Co., Ltd.

### 2.13. Western Blotting

Total protein was extracted using RIPA lysis buffer (Solarbio, R0010, Beijing, China). Proteins were denatured, separated by SDS-PAGE, and transferred to nitrocellulose membranes. The blots were probed with primary antibodies against Occludin (Proteintech, 27260-1-AP, Wuhan, China), ZO-1 (Proteintech, 21773-1-AP, Wuhan, China), E-cadherin (BD Biosciences, 610182, NJ, USA), β-catenin (Cell Signaling Technology, 8480S, MA, USA), and β-Actin (Sigma-Aldrich, A5441, MO, USA), followed by HRP-conjugated secondary antibodies. Protein bands were visualized using a gel imaging system.

### 2.14. Antibiotic Depletion Experiment

Three-week-old mice were fed a 0.4% GLSP or CON diet (*n* = 10 per group) for 10 weeks, then reallocated into four groups (*n* = 5 each): CON, GLSP, CON + ABX, and GLSP + ABX. The two ABX groups received 0.2 mL/day of an antibiotic cocktail by gavage for 2 weeks; the cocktail contained 1 g/L neomycin (MREDA, M1126-10g, Beijing, China), 1 g/L ampicillin (Konoscience, P001474, Beijing, China), 1 g/L metronidazole (Konoscience, B057154, Beijing, China), and 0.5 g/L vancomycin (Konoscience, AB105396, Beijing, China). All mice were then given 2.5% DSS in drinking water for 7 days to induce intestinal barrier damage.

### 2.15. Fecal Microbiota Transplantation (FMT)

Fecal microbiota suspension was prepared daily from ~300 mg of feces from GLSP-fed donor mice (*n* = 10) by suspending samples in 3 mL water, centrifuging (1500 rpm, 4 °C), and filtering through a 70 μm mesh. After acclimation, 6-week-old recipients received 0.2 mL of ABX cocktail by gavage daily for 14 days to establish a pseudo-germ-free state (>90% depletion by 16S qPCR), then were randomized into CON (*n* = 5), CON + DSS (*n* = 7), and FMT + DSS (*n* = 8). The FMT + DSS group was gavaged with 0.2 mL of the suspension daily for 7 days, while CON and CON + DSS continued ABX; finally, CON + DSS and FMT + DSS received 2.5% DSS in drinking water for 7 days to induce intestinal barrier injury.

### 2.16. Fecal Total Bacterial Load Was Measured by qPCR

Total microbial genomic DNA was extracted from mouse fecal samples using a fecal DNA extraction kit (Accurate Biotechnology, AG21036, Hunan, China) according to the manufacturer’s instructions. DNA integrity was assessed by 1% agarose gel electrophoresis, and concentration and purity were determined using a NanoDrop 2000 (Thermo Scientific). Quantitative PCR was performed with primers 27F (5′-AGAGTTTGATCCTGGCTCAG-3′) and 1492R (5′-TACGGCTACCTTGTTACGACTT-3′) targeting the bacterial 16S rRNA gene V3 region. Bacterial clearance efficacy following antibiotic treatment was evaluated by comparing copy numbers, with a reduction of ≥90% considered effective.

### 2.17. Statistical Analysis

All statistical analyses were performed with GraphPad Prism (San Diego, CA, USA), and significance was set at *p* < 0.05. Student’s *t*-test and one-way/two-way ANOVA with Tukey’s HSD post hoc test were applied for normally distributed data depending on whether two or three or more groups were compared, respectively. Conversely, for non-normally distributed data, the Wilcoxon test (two groups) and the Kruskal–Wallis test with Dunn’s post hoc test (three or more groups) were used.

## 3. Results

### 3.1. GLSP Diet Physiologically Regulates Intestinal Stem and Secretory Cells Activity

**Figure 1:**
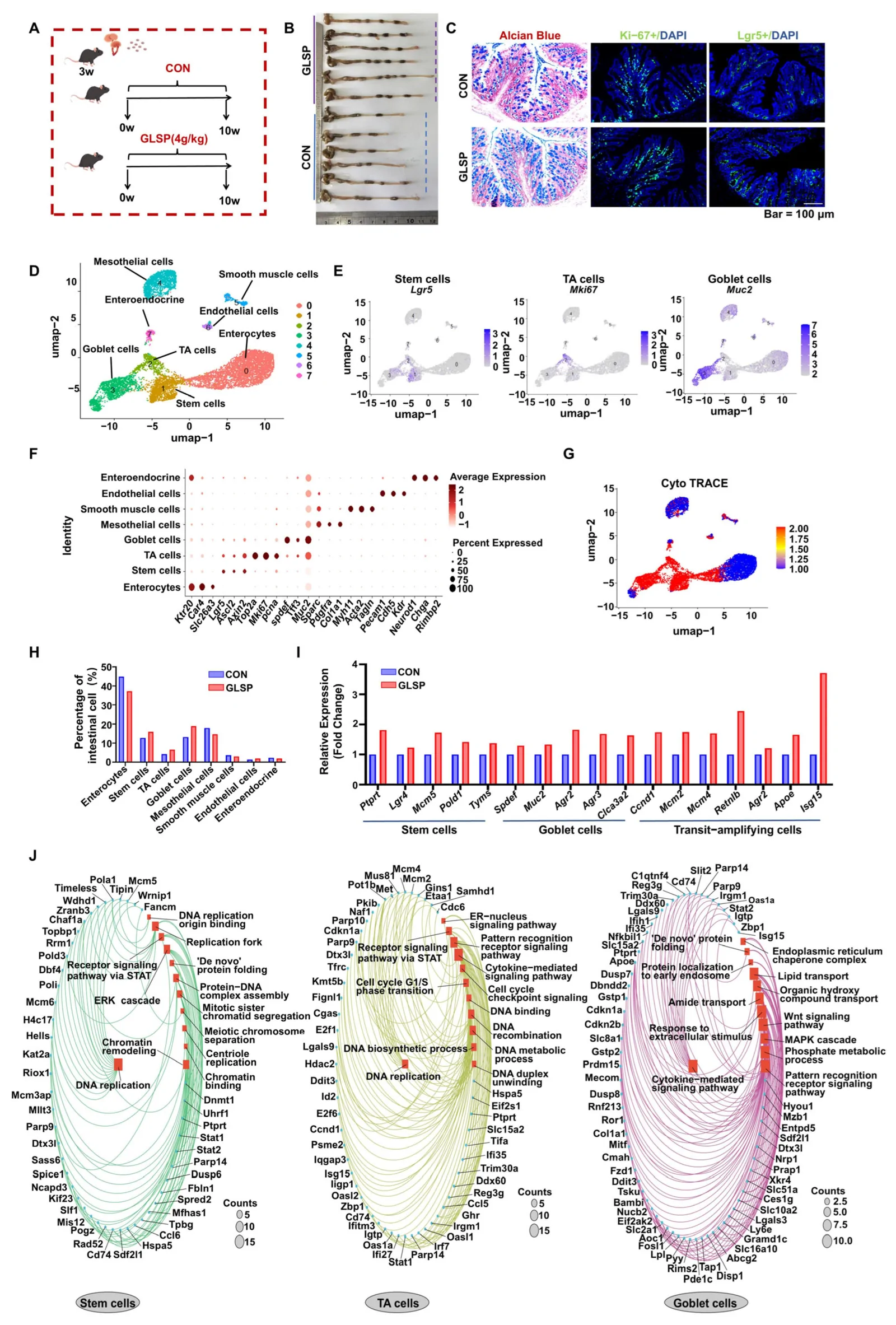
A GLSP diet physiologically regulates intestinal stem and secretory cells activity. (**A**) A schematic diagram of the animal experimental design. (**B**) Representative images of the colonic tissues (*n* = 6). (**C**) Representative images of colonic sections stained with Alcian blue, alongside immunofluorescent staining for Ki-67 and Lgr5 (*n* = 6). (**D**) A UMAP plot of epithelial cells from colon tissue, showing 8 main clusters. (**E**) UMAP plots showing the expression levels of signature genes in different clusters. (**F**) A bubble heatmap depicting the expression levels of selected marker genes for each cluster. (**G**) A distribution plot of single-cell differentiation potential. (**H**) The proportional distribution of various cell types within the clusters. (**I**) A statistical table of differentially expressed genes (clusters 1, 2, and 4). (**J**) A functional enrichment analysis based on single-cell RNA sequencing data. Scale bar, 100 μm.

To investigate the effects of GLSP on intestinal structure and function, 3-week-old male mice were randomly divided into the control (CON) group and GLSP-diet group. After 10 weeks of feeding, colon tissues were collected for subsequent analyses (Figure 1A). The GLSP-diet group exhibited increased colon length (Figure 1B and Figure S1A). Alcian blue staining suggested an increase in the number of goblet cells in the GLSP-diet group. Immunofluorescence staining revealed a trend toward increased expression of intestinal stem cell marker LGR5 and the proliferation marker Ki-67 (Figure 1C and Figure S1B), which suggested that a GLSP diet may facilitate intestinal epithelial renewal. To further clarify the underlying mechanisms, single-cell sequencing was performed on the two sets of colon samples (Figure S1C). Immune cells and epithelial cells were first distinguished using Ptprc as a marker gene (Figure S1D), and the epithelial cells were subsequently classified into eight subsets (Figure 1D). Each cluster was identified based on characteristic genes and visualized using UMAP plots and bubble plots (Figure 1E,F and Figure S1E). The differentiation status and annotation accuracy were validated using the CytoTRACE algorithm (Figure 1G). Notably, the proportions of stem cells, transit-amplifying (TA) cells, and goblet cells were significantly increased in the GLSP-diet group (Figure 1H). Based on differentially expressed gene analysis, a significant upregulation was observed in functionally related genes of the three cell populations mentioned above (Figure 1I). GO and KEGG enrichment analysis further showed that DNA replication pathways were enriched in stem cells, proliferation-related pathways in TA cells, and protein synthesis pathways in goblet cells (Figure 1J). These findings suggested that a GLSP diet may promote rapid renewal of the intestinal epithelium by activating intestinal stem cells, promoting TA cell proliferation, and upregulating mucin synthesis in goblet cells, thereby potentially strengthening intestinal barrier function.

### 3.2. GLSP Diet Promotes the Restoration of Intestinal Barrier Function and Alleviates DSS-Induced Intestinal Epithelial Damage

Given the robust homeostatic capacity of healthy organisms, the regulatory effect of a GLSP diet may be limited under normal physiological conditions. However, during natural aging, the regenerative capacity of intestinal cells declines, and intestinal barrier function is gradually impaired. Therefore, this study used 8-month-old mice as a model for intervention (Figure S2). The results indicated that not only can a GLSP diet promote proliferation activity and goblet cell function, it also effectively restores junction-associated proteins, thereby enhancing the intestinal epithelial mechanical barrier and delaying the process of intestinal aging.

**Figure 2:**
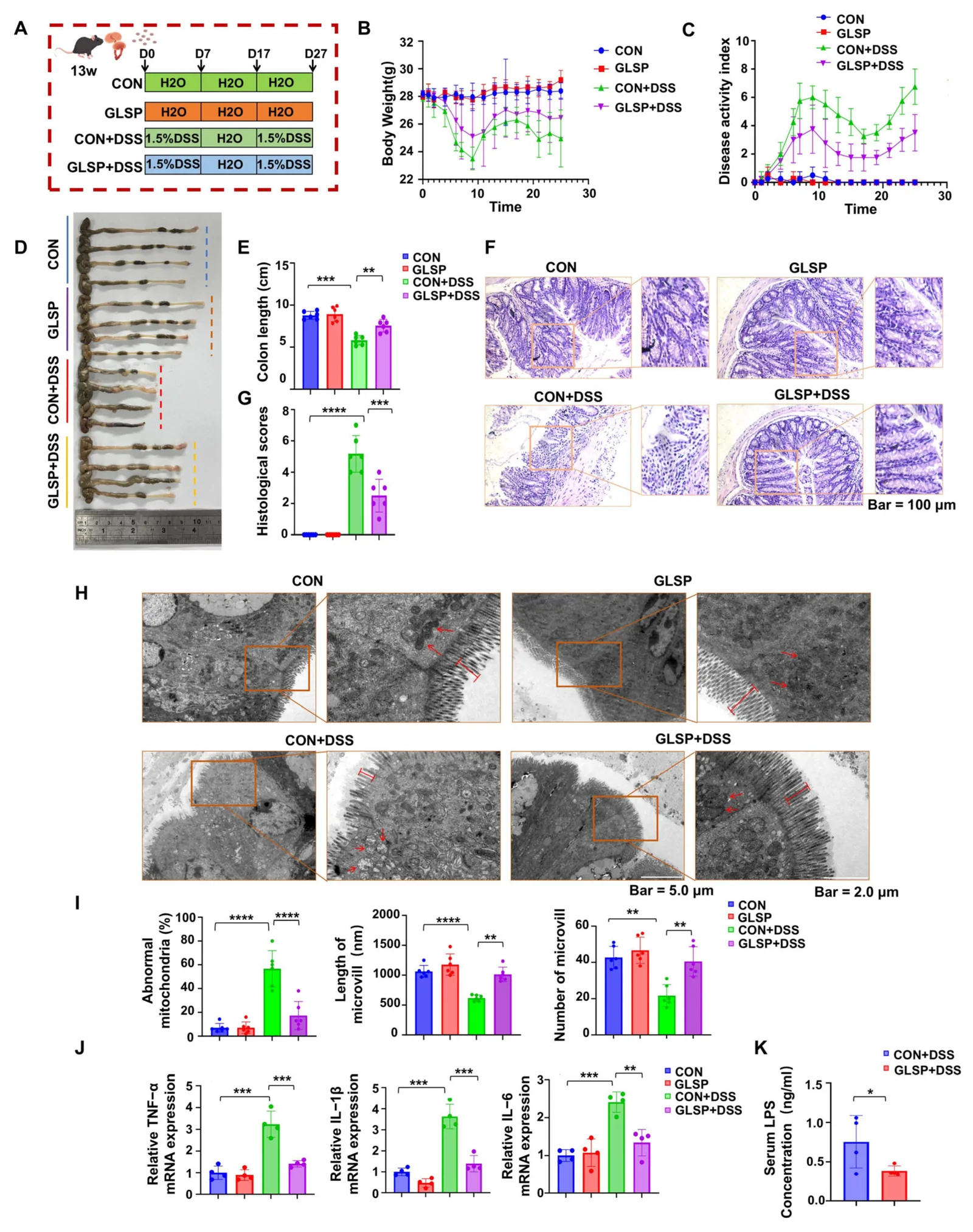
The GLSP diet alleviates DSS-induced intestinal epithelial damage. (**A**) A schematic diagram of the animal experimental design. (**B**) The body weight change of the mice (*n* = 6). (**C**) The DAI score (*n* = 6). (**D**,**E**) Representative images of colonic tissues and statistical analysis of colonic length (*n* = 6). (**F**) Representative images of colonic sections stained with H&E (*n* = 6). (**G**) The histological scores (*n* = 6). (**H**) Representative TEM images of colonic tissues (*n* = 6). (**I**) The number of microvilli, length of microvilli, and percentage of abnormal mitochondria (*n* = 6). (**J**) The levels of pro-inflammatory cytokines (TNF-α, IL-1β, IL-6) in colonic tissues (*n* = 4). (**K**) The serum levels of LPS (*n* = 4). * *p* < 0.05, ** *p* < 0.01, *** *p* < 0.001, **** *p* < 0.0001. Scale bar, 100 μm, 5.0 μm or 2.0 μm.

**Figure 3:**
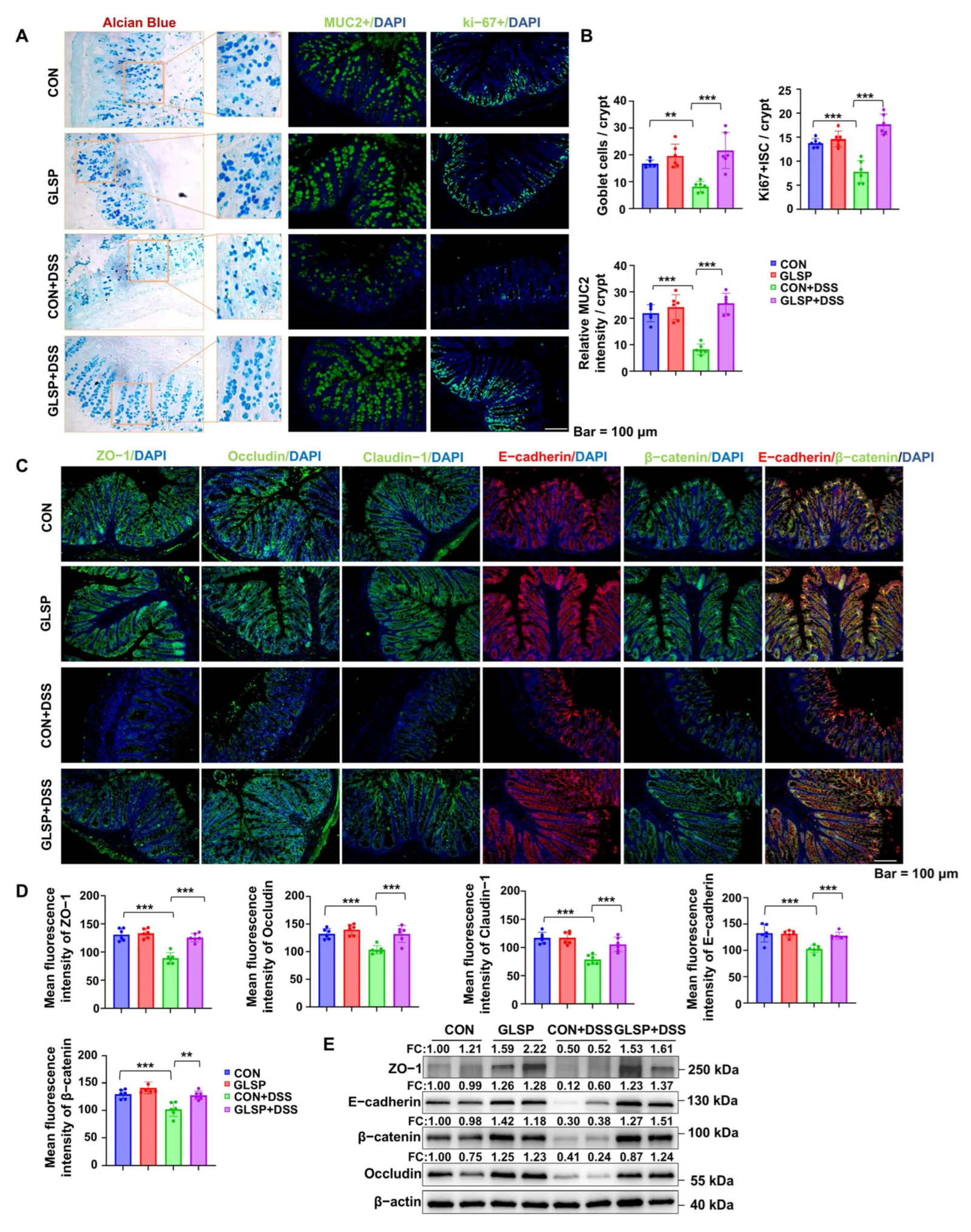
The GLSP diet promotes intestinal cell proliferation and repairs both the mucosal barrier and physical barrier. (**A**) Representative images of colonic sections stained with Alcian blue, alongside immunofluorescent staining for MUC2 and Ki-67 (*n* = 6). (**B**) The number of goblet cells and relative expression levels of Ki-67 and MUC2 per crypt (*n* = 6). (**C**,**D**) Representative immunofluorescence images and mean fluorescence intensity of ZO-1, Occludin, Claudin-1, E-cadherin, and β-catenin in colonic sections (*n* = 6). (**E**) Western blot analysis of ZO-1, E-cadherin, β-catenin and Occludin in colonic tissues. The band intensities were normalized to β-actin, and the relative expression (Fold change, FC) refers to the ratio relative to the control group. ** *p* < 0.01, *** *p* < 0.001. Scale bar, 100 μm.

To further investigate the role of the GLSP diet in maintaining intestinal homeostasis, we utilized a DSS-induced mice model (a classic model of intestinal barrier damage) for the experiments (Figure 2A). Compared with the CON group, mice in the DSS group exhibited significant body weight loss and a marked increase in disease activity index (DAI) scores. The GLSP diet effectively alleviated weight loss, improved symptoms of diarrhea and hematochezia, and significantly reduced DAI scores (Figure 2B,C). In addition, DSS induced severe colonic pathological damage, which was characterized by a significant reduction in colon length, destruction of crypt structure, loss of mucosal integrity, and increased histological scores (Figure 2D–G). TEM further revealed ultrastructural damage in the DSS group, including increased tight junction gaps, reduced and shortened microvilli, and mitochondrial damage (Figure 2H,I). In contrast, the GLSP diet significantly reversed the pathological and ultrastructural alterations and reduced the histological scores. Moreover, the GLSP diet effectively downregulated the DSS-induced elevated mRNA expression of TNF-α, IL-1β and IL-6 in the colon (Figure 2J), and reduced serum LPS levels (Figure 2K). Further investigation revealed that the number of goblet cells and the secretion of MUC2 significantly decreased in the DSS model group, whereas these parameters markedly increased in the GLSP-diet group, indicating that GLSP can improve mucin synthesis and secretion. The immunofluorescence results showed that the expression of Ki-67 protein in colonic tissues of the GLSP-diet group exhibited an upward trend, demonstrating enhanced cell proliferation activity (Figure 3A,B). In addition, the GLSP diet significantly reversed the DSS-induced downregulation of tight junction proteins (Claudin-1, Occludin, ZO-1) and adherens junction proteins (E-cadherin, β-catenin) (Figure 3C–E). In the acute DSS model, we also observed a similar protective effect of GLSP on the intestinal barrier (Figure S3). In summary, the GLSP diet exerts a dual protective role in DSS-induced intestinal injury: on one hand, it promotes cell proliferation and increases goblet cell numbers and MUC2 expression to repair the mucus barrier; on the other hand, it upregulates junction proteins to strengthen the physical barrier. These two mechanisms act synergistically to promote the restoration and reconstruction of intestinal barrier function.

### 3.3. Gut Microbiota Depletion Abolishes GLSP-Mediated Maintenance of Intestinal Homeostasis

**Figure 4:**
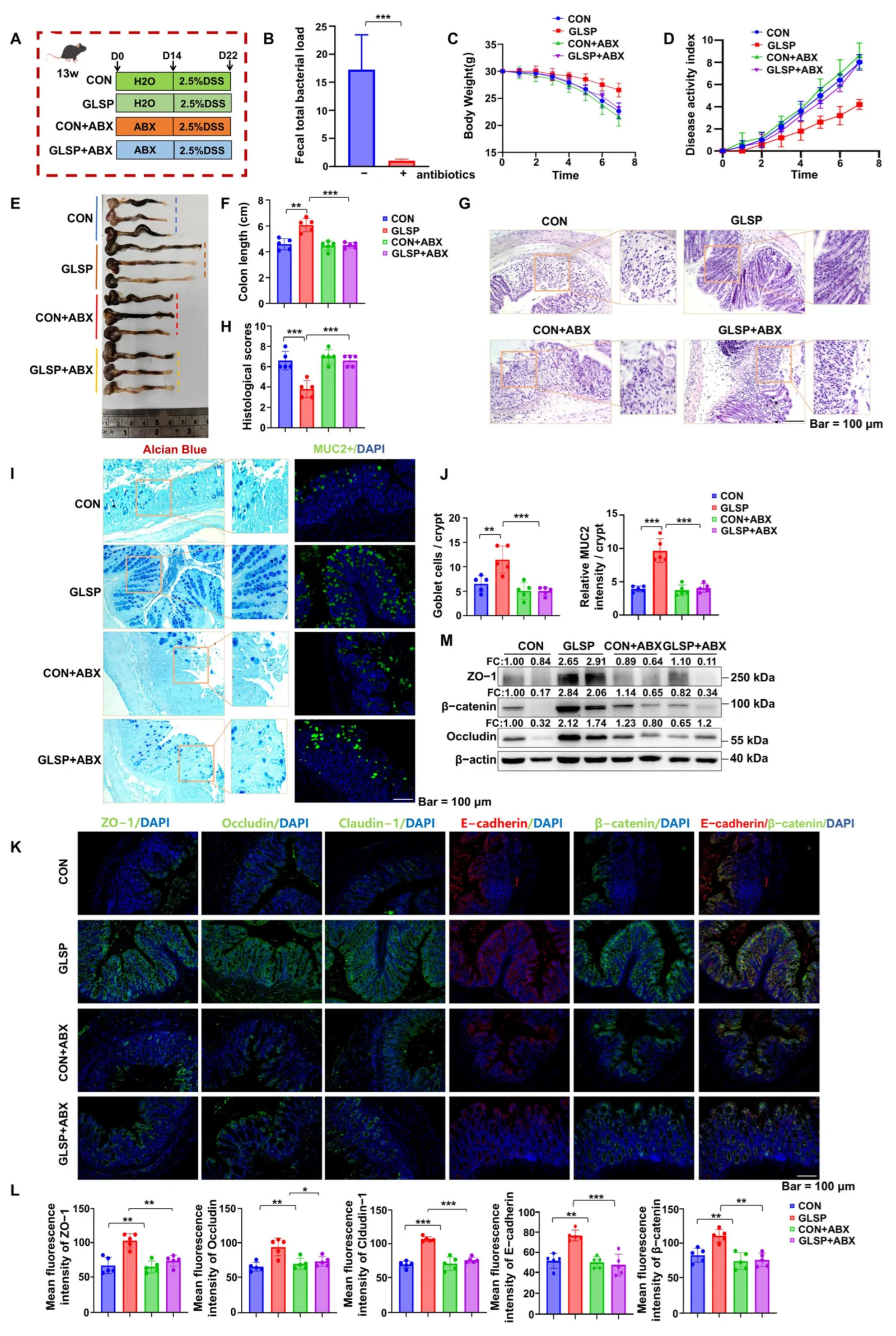
Gut microbiota depletion abolishes GLSP-mediated maintenance of intestinal homeostasis. (**A**) A schematic diagram of the animal experiment. (**B**) The fecal total bacterial load measured by qPCR (*n* = 10). (**C**) The body weight change of the mice (*n* = 5). (**D**) The DAI score (*n* = 5). (**E**,**F**) Representative images of colonic tissues and colonic length (*n* = 5). (**G**,**H**) Images of colonic sections stained with H&E and histological scores (*n* = 5). (**I**) Representative colonic sections stained with Alcian blue, alongside immunofluorescent staining for MUC2. (**J**) The number of goblet cells and relative expression levels of MUC2 (*n* = 5). (**K**,**L**) Representative immunofluorescence and mean fluorescence intensity of ZO-1, Occludin, Claudin-1, E-cadherin, and β-catenin in colonic sections (*n* = 5). (**M**) Western blot analysis of ZO-1, E-cadherin, β-catenin and Occludin in colonic tissues. The band intensities were normalized to β-actin, and the relative expression (Fold change, FC) refers to the ratio relative to the control group. * *p* < 0.05, ** *p* < 0.01, *** *p* < 0.001. Scale bar, 100 μm.

The gut microbiota plays a central role in maintaining colonic homeostasis. Given that GLSP diet can reach the colon after oral administration and may modulate the gut microbiota, and that its poorly absorbed active components are more likely to act as metabolic substrates for the microbiota, we hypothesized that the GLSP diet exerts its protective effects in a microbiota-associated manner. To test this, we first established a microbiota-depleted mouse model through intragastric administration of ABX (Figure 4A). The total bacterial load was measured via fecal PCR, which confirmed microbiota depletion (Figure 4B). Compared with the GLSP-diet group, mice in the GLSP-diet with antibiotics (GLSP + ABX) group exhibited a significant decrease in body weight, an increase in DAI scores, and shortened colon length (Figure 4C–F). Their phenotype was more consistent with that of the CON and the CON with antibiotics (CON + ABX) groups. Moreover, the colonic mucosa in the GLSP + ABX group showed severe structural damage, extensive infiltration of inflammatory cells, and significantly elevated pathological scores (Figure 4G,H). Alcian blue and immunofluorescence staining further revealed a significant reduction in goblet cell counts and MUC2 secretion (Figure 4I,J). Additionally, the expression of tight junction proteins (Claudin-1, Occludin, ZO-1) and adherens junction proteins (E-cadherin, β-catenin) were significantly downregulated (Figure 4K–M). In summary, the GLSP diet failed to improve DSS-induced colonic damage after the depletion of the gut microbiota, suggesting that its protective effect on the intestinal barrier may depend on the gut microbiota.

### 3.4. GLSP Diet Reshaped the Gut Microbiota of Mice and Concurrently Increased the Relative Abundance of Probiotics

**Figure 5:**
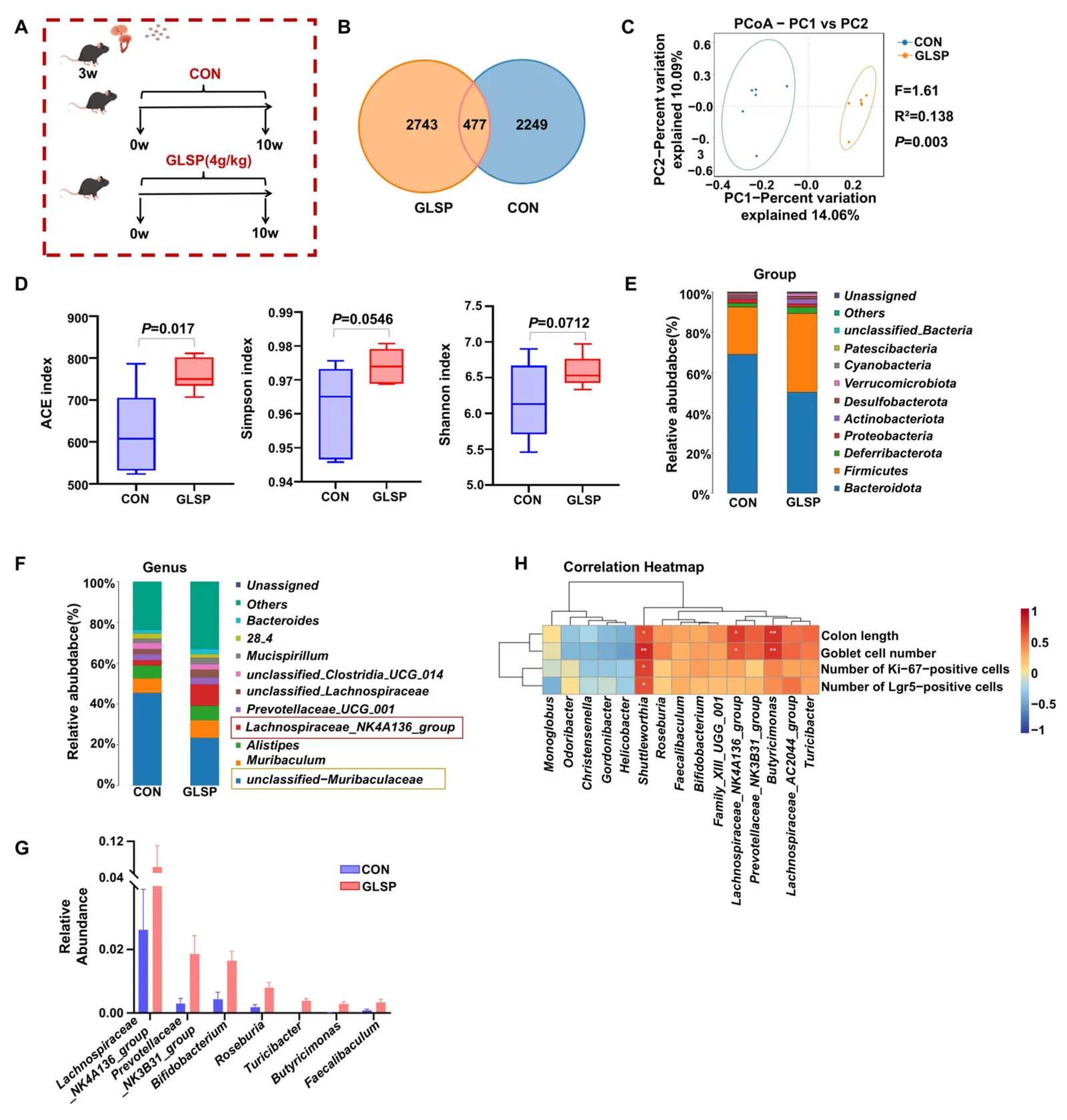
The GLSP diet reshaped the gut microbiota of the mice and concurrently increased the relative abundance of probiotics. (**A**) A schematic diagram of the animal experiment. (**B**) A Venn diagram of features (*n* = 6). (**C**) A PCA plot (*n* = 6). (**D**) The alpha diversity of mouse gut microbiota: ACE index, Simpson index, and Shannon index (*n* = 6). (**E**,**F**) The relative abundance of species at the phylum and genus levels (*n* = 6). (**G**) An ANOVA analysis based on relative abundance at the genus level (*n* = 6). (**H**) A heatmap of correlations between gut microbiota and colon tissue parameters (Spearman correlation analysis).

To unveil the regulatory mechanisms of the gut microbiota by the GLSP diet, we performed 16S rRNA gene sequencing on fecal specimens (Figure 5A). Rarefaction curves indicated that the sequencing depth was sufficient and the data were reliable (Figure S4A). The Venn diagram illustrates the shared and unique features between the two groups (Figure 5B). PCA, UPGMA, and clustering heatmap analyses revealed significant differences in microbial community in the GLSP-diet and CON groups, with each group forming distinct clusters (Figure 5C and Figure S4B,C). Alpha diversity analysis showed that the GLSP diet significantly increased the ACE, Simpson, and Shannon indices, indicating enhanced microbial richness and diversity (Figure 5D). Rank abundance curve further confirmed that species richness and evenness were higher in the GLSP-diet group than in the CON group (Figure S4D). Together, these findings indicate that a GLSP diet can effectively reshape the gut microbiota. Phylum-level analysis revealed that Firmicutes and Bacteroidetes were the dominant phyla across all samples (Figure 5E). Genus-level analysis indicated that the GLSP diet significantly increased the abundance of several health-associated probiotic genera, including *Lachnospiraceae_NK4A136_group*, *Prevotellaceae_NK3B31_group*, *Bifidobacterium*, *Roseburia*, *Butyricimonas*, and *Faecalibaculum* (Figure 5F,G). Notably, active metabolites such as short-chain fatty acids and lactic acid produced by the intestinal microflora can influence intestinal epithelial renewal and repair by regulating the proliferation and differentiation of intestinal cells [20,21,22,23,24,25,26,27,28,29,30,31,32,33,34,35]. Moreover, the relative abundances of these genera were significantly positively correlated with the number of colonic goblet cells, the number of stem cells, and colon length (Figure 5H). These findings suggest that the GLSP diet may maintain intestinal homeostasis by remodeling the gut microbiota (enriching health-associated genera) and their metabolites, which indirectly promote epithelial regeneration and barrier repair.

### 3.5. Gut Microbiota Is Essential for GLSP-Mediated Intestinal Homeostasis

**Figure 6:**
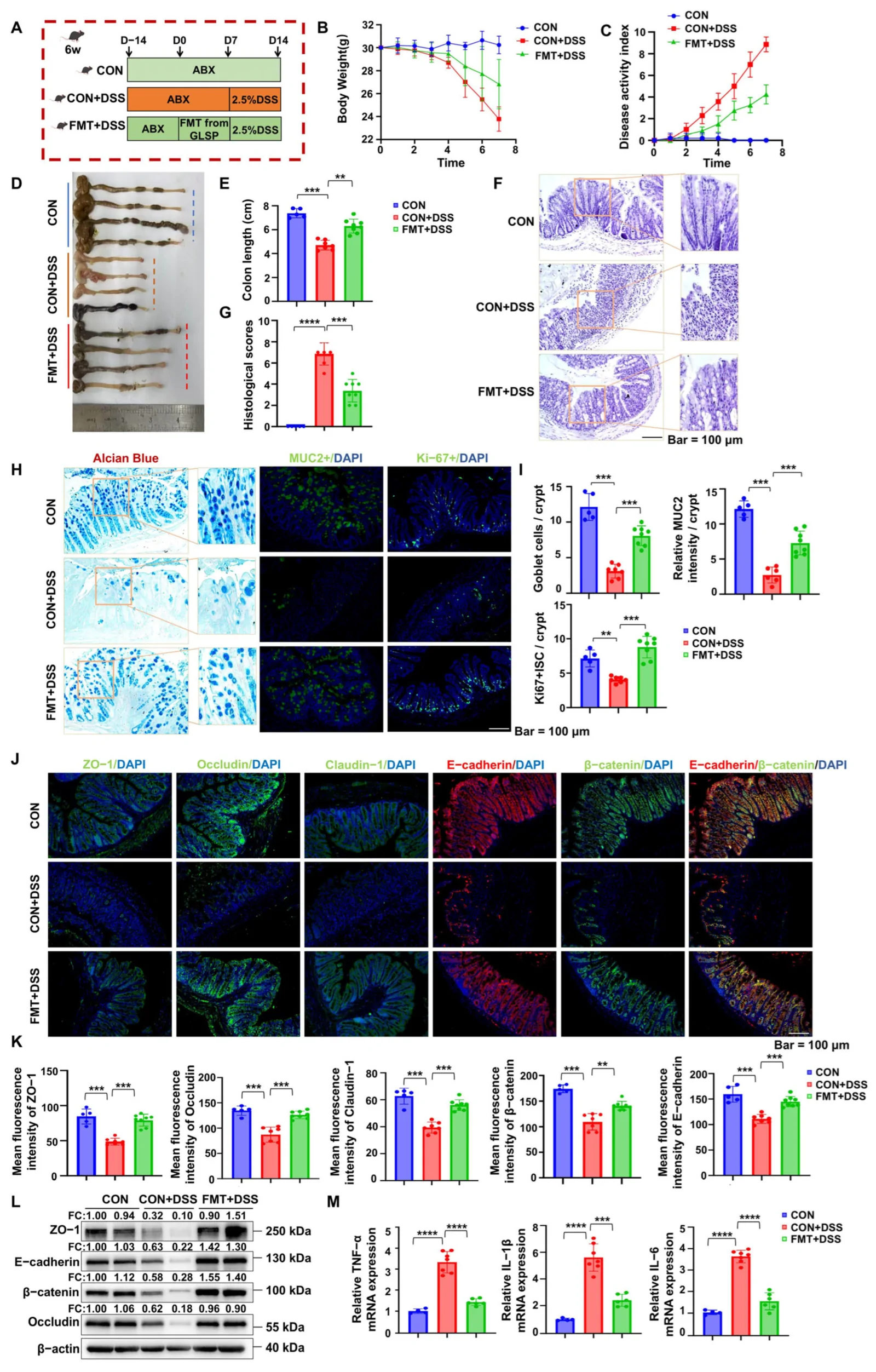
Gut microbiota is essential for GLSP-mediated intestinal homeostasis. (**A**) A schematic diagram of the animal experiment. (**B**) The body weight change of the mice (*n* = 5/7/8). (**C**) The DAI score (*n* = 5/7/8). (**D**,**E**) Representative images and length of colonic tissues (*n* = 5/7/8). (**F**,**G**) Representative images and histological scores of colonic sections stained with H&E (*n* = 5/7/8). (**H**) Colonic sections stained with Alcian blue, alongside immunofluorescent staining for MUC2 and Ki-67. (**I**) The number of goblet cells and relative expression levels of MUC2 Ki-67 (*n* = 5/7/8). (**J**,**K**) Representative immunofluorescence images and mean fluorescence intensity of ZO-1, Occludin, Claudin-1, E-cadherin, and β-catenin in colonic sections (*n* = 5/7/8). (**L**) Western blot analysis of ZO-1, E-cadherin, β-catenin and Occludin in colonic tissues. The band intensities were normalized to β-actin, and the relative expression (Fold change, FC) refers to the ratio relative to the control group. (**M**) An analysis of the pro-inflammatory cytokines (TNF-α, IL-1β, IL-6) in colonic tissues (*n* = 4/7/6). ** *p* < 0.01, *** *p* < 0.001, **** *p* < 0.0001. Scale bar, 100 μm.

To verify the critical role of these differential floras in the maintenance of intestinal homeostasis by the GLSP diet, we further conducted an FMT experiment (Figure 6A). FMT treatment significantly attenuated weight loss, alleviated diarrhea and hematochezia, and reduced the DAI score in DSS-treated mice (Figure 6B,C). Moreover, the DSS-induced colon shortening was significantly reversed in the FMT group (Figure 6D,E). HE staining revealed severe disruption of the colonic mucosal structure, significant inflammatory cell infiltration, and higher histological scores in the DSS model group. In contrast, these pathological changes were significantly attenuated in the FMT group (Figure 6F,G). In addition, the number of proliferating cells and goblet cells, as well as MUC2 secretions were all reduced in the DSS model group, whereas FMT treatment significantly reversed these abnormal changes (Figure 6H,I). Concurrently, the impaired intestinal barrier function in DSS mice was markedly restored, as evidenced by the upregulated expression of tight junction proteins (Claudin-1, Occludin, ZO-1) and adherens junction proteins (E-cadherin, β-catenin) (Figure 6J–L). FMT also significantly reduced the mRNA expression of pro-inflammatory cytokines TNF-α, IL-1β, and IL-6 in colon tissues (Figure 6M). In summary, gut microbiota plays a critical role in mediating the protective effects of the GLSP diet on intestinal homeostasis.

### 3.6. GLSP Diet Remodels Metabolites of Gut Microbiota in Mice

**Figure 7:**
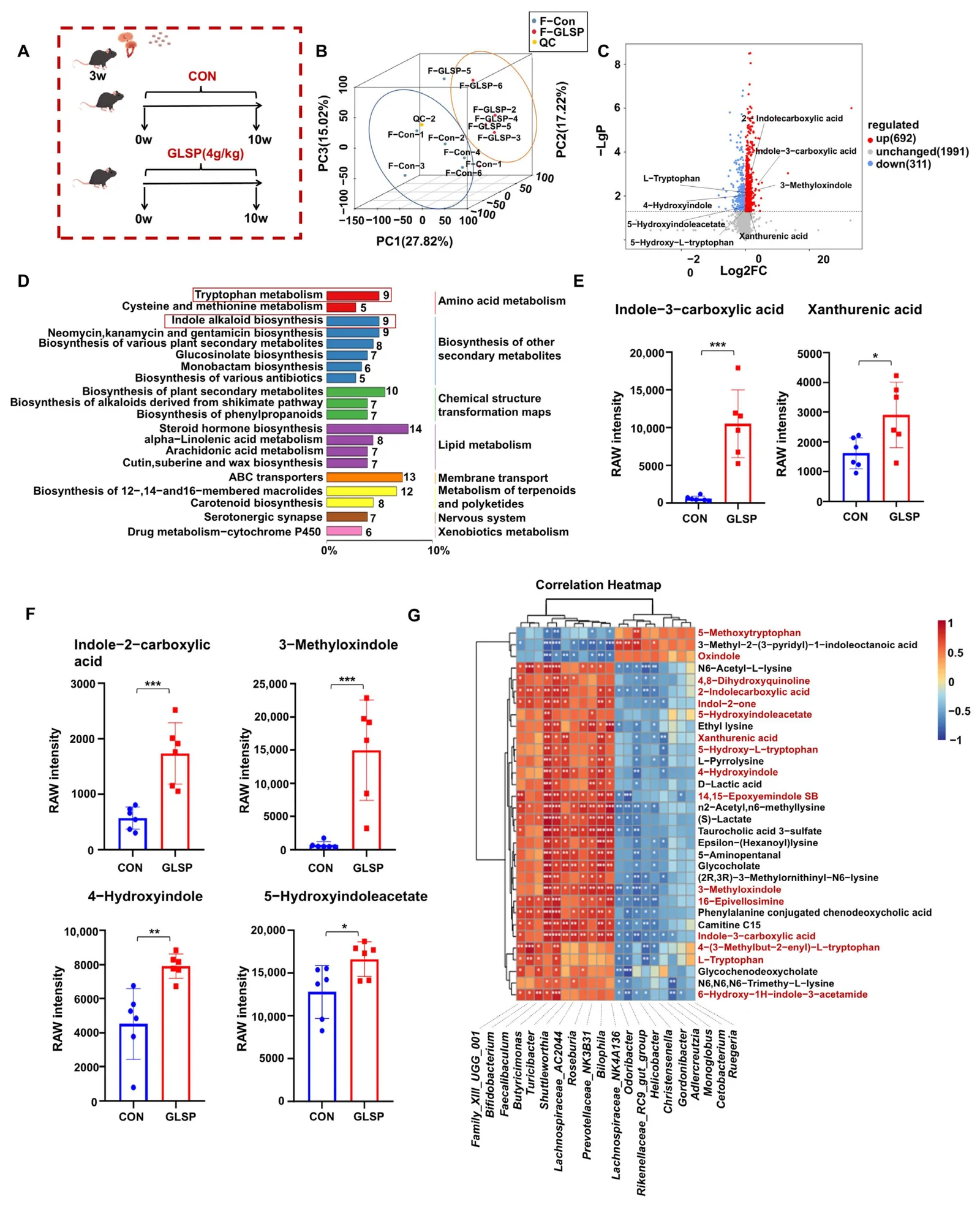
The GLSP diet remodels metabolites of gut microbiota in mice. (**A**) A schematic diagram of the animal experiment (*n* = 6). (**B**) A principal component analysis (PCA) plot for the difference-based grouping (*n* = 6). (**C**) A volcano plot of differential metabolites (*n* = 6). (**D**) A KEGG pathway enrichment analysis plot (*n* = 6). (**E**,**F**) A comparison of the relative expression levels of key tryptophan metabolites (*n* = 6). (**G**) A Spearman correlation heatmap between differential microbiota and key metabolites (Spearman correlation analysis). * *p* < 0.05, ** *p* < 0.01, *** *p* < 0.001.

Given the regulatory effects of the GLSP diet on the gut microbiome, we further performed untargeted metabolomics to identify functional microbial metabolites (Figure 7A and Figure S5). PCA and volcano plot analyses revealed that the GLSP diet significantly altered the gut metabolome, with most differential metabolites being tryptophan and its indole derivatives (Figure 7B,C and Figure S5E). KEGG pathway enrichment analysis further confirmed that the GLSP diet markedly affected multiple gut microbial metabolic pathways, including amino acid metabolism, lipid metabolism, and the biosynthesis of other secondary metabolites (Figure 7D). Among these, tryptophan metabolism and indole derivative biosynthesis may serve as the predominant pathways modulated by the GLSP diet. Given that tryptophan metabolites, particularly indole derivatives, play a critical role in maintaining intestinal barrier function and modulating anti-inflammatory responses [36], we performed quantitative analysis of the significantly altered indole derivatives. The results showed that, in addition to the previously reported indole derivatives associated with intestinal function (indole-3-carboxylic acid, xanthurenic acid, and 5-Hydroxyindoleacetic acid [37,38,39]), the levels of other indole metabolites with previously unknown functions—such as indole-2-carboxylic acid, 3-Methyloxindole, and 4-Hydroxyindole—were also significantly upregulated (Figure 7E,F). Furthermore, correlation analysis between the microbiota and metabolites revealed significant associations between multiple differentially abundant microbial taxa and various altered metabolites, including tryptophan, lactic acid, and bile acid metabolites (Figure 7G). Notably, the levels of certain indole metabolites were positively correlated with genera such as the *Lachnospiraceae NK4A136 group* and *Butyricimonas*. Collectively, these findings suggest that the GLSP diet may exert its intestinal protective effects by modulating gut microbial metabolites, particularly through the regulation of tryptophan metabolism and the production of indole derivatives.

## 4. Discussion

**Figure 8:**
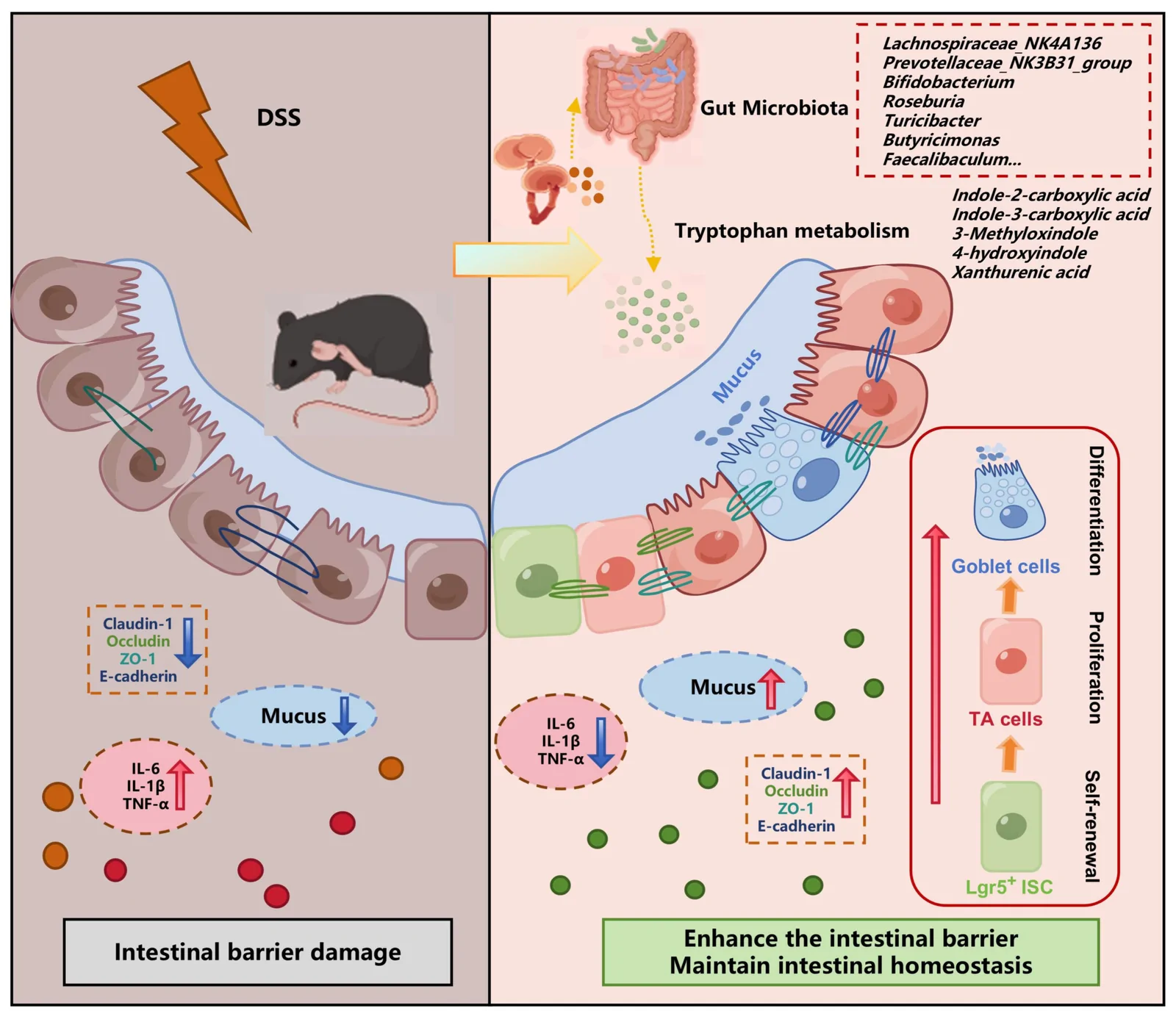
Mechanism diagram of *Ganoderma lucidum* spore powder in maintaining intestinal homeostasis.

In the present study, we systematically elucidated the protective effects of a GLSP diet on intestinal barrier function and delineated a microbiota-associated mechanism involving tryptophan metabolism and indole derivative production. Our findings demonstrate that the GLSP diet promotes epithelial renewal under physiological conditions, and potently alleviates DSS-induced colitis through coordinated reinforcement of mucus and physical barriers. Notably, these protective effects are entirely contingent upon an intact gut microbial community. Collectively, our results indicate that the GLSP diet promoted intestinal homeostasis by modulating the microbiota–metabolite–epithelium axis (Figure 8).

Under homeostatic conditions, the GLSP diet primes the intestinal epithelium for enhanced turnover capacity. This was supported by a trend toward increased colonic length, augmented ISC (LGR5^+^) and proliferative (Ki-67^+^) cell populations, and expanded goblet cell numbers, as revealed by histology and single-cell sequencing. Notably, tight and adherens junction protein expression remained unaltered in healthy mice, consistent with the concept that homeostatic epithelium already maintains robust junctional integrity, leaving limited room for further enhancement. This paradigm aligns with previous reports that dietary interventions often manifest more pronounced effects under challenge conditions than in steady-state physiology. In DSS-induced injury, the GLSP diet robustly restored Occludin, ZO-1, Claudin-1, E-cadherin, and β-catenin, underscoring its capacity to reinforce the physical barrier. These findings are consistent with recent reports demonstrating that *Ganoderma lucidum* spore polysaccharides significantly restore intestinal barrier integrity and upregulate the expression of barrier proteins including ZO-1, Occludin, and MUC2 in colitis models [17].

The functional relevance of GLSP-diet-induced changes in ISC and TA cell compartments warrants consideration. Single-cell RNA sequencing revealed that the GLSP diet upregulated DNA replication and proliferation-associated pathways in stem and TA cells, while goblet cells showed enrichment of protein synthesis. This coordinated enhancement of epithelial proliferation and secretory differentiation likely underpins the accelerated barrier restitution observed after DSS injury. These findings echo recent work demonstrating that microbial metabolites, particularly SCFAs, stimulate ISC proliferation through GPCR-mediated pathways and histone deacetylase inhibition [40]. Our microbiota data revealed that the GLSP diet enriched SCFA-producing genera, including *Lachnospiraceae_NK4A136_group*, *Prevotellaceae_NK3B31_group*, *Roseburia*, and *Butyricimonas*, providing a potential mechanistic link between microbial shifts and epithelial dynamics [21,24,25,29,31]. This is consistent with studies showing that *Ganoderma lucidum* spore oligosaccharides can be effectively utilized by gut microbiota, significantly promoting the production of major SCFAs (acetate, propionate, and butyrate), and that water-soluble polysaccharides from *Ganoderma lucidum* spores increase SCFA production and improve intestinal barrier function [17,41,42].

A critical finding of this study is the absolute dependence of the GLSP diet’s protective effects on the gut microbiota. Antibiotic-mediated depletion completely abolished the GLSP diet’s capacity to mitigate DSS-induced pathology, reverse junctional protein loss, or restore goblet cell function. This observation demonstrates that the beneficial effects of GLSP-diet actions are not mediated by the host epithelium alone, but rather require gut microbial intermediaries for metabolic conversion. This is consistent with the physicochemical properties of GLSP—as a high-molecular-weight polysaccharide that resists host digestion and reaches the colon as a fermentation substrate [41,43,44]. Our 16S rRNA sequencing data confirmed that the GLSP diet profoundly reshaped microbial community structure, increasing alpha diversity and promoting the expansion of multiple probiotic genera with known metabolic capabilities. Among these, *Bifidobacterium* and *Lachnospiraceae* members are well-established producers of lactate and butyrate, respectively, which exert trophic effects on colonic epithelia [21,27]. The positive correlations between these genera and key phenotypic parameters (goblet cell count, stem cell activity, and colonic length) reinforce the functional significance of these microbial shifts.

To establish causality, we performed FMT from GLSP-diet donors into DSS-challenged recipients. The transfer of GLSP-shaped microbiota successfully recapitulated the protective phenotype, including reduced injury severity, preserved epithelial architecture, restored junctional proteins, and increased goblet cell populations. These results unequivocally demonstrate that the gut microbiota serves as a sufficient mediator of the GLSP diet’s intestinal protective effects. Moreover, FMT reduced circulating inflammatory cytokines, suggesting that microbiota-associated barrier reinforcement limits systemic endotoxin exposure and consequently attenuates systemic inflammatory. This interpretation is further supported by studies demonstrating that fecal microbiota transplantation from *Ganoderma lucidum* spore polysaccharide-treated donors confers protection against intestinal barrier impairment [17].

Furthermore, it should be noted that although antibiotic depletion experiments are widely employed to establish microbiome dependency, broad-spectrum antibiotics may exert direct effects on the host beyond their antimicrobial activity. For instance, antibiotics can modulate host immune responses and intestinal epithelial function, thereby potentially affecting barrier integrity independently of microbiota depletion. Consequently, although our antibiotic depletion experiments, together with the FMT experiments, collectively support that the effects of GLSP are microbiota-associated, residual direct effects of antibiotics cannot be entirely excluded as partially contributing to the observed phenotypes. Future investigations employing germ-free mice or more selective microbiota-targeting approaches will facilitate further elucidation of the specific contributions of the microbiota.

Untargeted metabolomics analysis provided further insight into the molecular mediators underlying the effects of GLSP. The GLSP diet profoundly remodeled the metabolic landscape of the intestinal lumen, with significant alterations in lysine metabolism, bile acid metabolism, lactate metabolism, and tryptophan metabolism, suggesting that the protective effects of the GLSP diet may involve a multi-metabolite network rather than a single pathway. The most prominently altered pathways in our metabolomic analysis are the tryptophan metabolism and indole derivative biosynthesis pathways. Indole derivatives represent a class of microbial metabolites with established roles in maintaining intestinal homeostasis, possibly through the aryl hydrocarbon receptor (AhR) pathway, which drives IL-22 production, enhances tight junction expression, and promotes mucin synthesis [45]. Beyond previously reported indole-3-carboxylic acid, xanthurenic acid, and 5-hydroxyindoleacetic acid, our metabolomic analysis identified indole-2-carboxylic acid, 3-Methyloxindole and 4-hydroxyindole as significantly elevated metabolites. The positive correlations between these indole metabolites and GLSP-enriched bacterial genera, particularly *Lachnospiraceae_NK4A136_group* and *Butyricimonas*, suggest that the GLSP diet may selectively foster taxa harboring tryptophan-metabolizing capabilities. Collectively, our findings highlight tryptophan–indole metabolism as a key mechanistic axis linking GLSP-shaped microbiota to intestinal barrier protection, while the coordinated alterations in lysine, bile acid, and lactate metabolism suggest a broader metabolic reprogramming that may act synergistically to preserve intestinal homeostasis.

Furthermore, it should be noted that although our multi-omics analysis identified several novel indole derivatives (such as indole-2-carboxylic acid, 3-Methyloxindole, and 4-hydroxyindole) that were significantly elevated following the GLSP diet, and these metabolites are closely associated with GLSP-enriched bacterial taxa and intestinal barrier protection, direct functional validation of these specific compounds remains to be completed. In this study, we did not perform direct in vitro or in vivo functional validation (e.g., cell monolayer barrier integrity assays or AhR reporter gene assays) to demonstrate that these individual indole derivatives directly activate the AhR signaling pathway or enhance tight junction expression. Therefore, although correlational and multi-omics evidence strongly supports the mechanistic association, future studies employing indole-2-carboxylic acid, 3-Methyloxindole, and 4-hydroxyindole in cellular and in vivo models are warranted to establish causality and determine whether these metabolites can be utilized for intestinal barrier protection.

It is important to acknowledge that GLSP, as used in this study, is a complex mixture rather than a chemically pure compound, and the specific bioactive fraction responsible for the observed effects remain to be identified. Nevertheless, this does not diminish the significance of our findings. Rather, we deliberately employ GLSP as a mechanistic tool to uncover the host–microbiota crosstalk underlying intestinal barrier maintenance. Our data unequivocally demonstrates that the protective outcome is strictly dependent on the gut microbiota and its metabolic output, particularly tryptophan-derived indole derivatives. This shifts the mechanistic focus from identifying a single active component to defining the microbial mediators and metabolic pathways that execute the protective effect. Building on this foundation, our ongoing studies aim to functionally validate the candidate taxa (e.g., *Lachnospiraceae_NK4A136_group*, *Bifidobacterium*, *Roseburia*) and the newly identified indole metabolites (indole-2-carboxylic acid) emerging from our multi-omics screening. Future studies will determine whether these bacterial strains can serve as next-generation probiotics and whether the identified indole derivatives can act as postbiotic effectors to promote intestinal homeostasis and host health. Thus, although GLSP remains a complex mixture, its value lies in illuminating a therapeutically targetable microbiota–metabolite axis that may be exploited with greater precision in future therapeutic applications.

## 5. Conclusions

In conclusion, this study reveals that the GLSP diet exerts its intestinal protective effects through a gut microbiota-associated mechanism, primarily by enriching SCFA-producing genera and promoting tryptophan metabolism to generate indole derivatives that reinforce epithelial barrier function. Collectively, these findings demonstrate that the GLSP diet prevents intestinal barrier dysfunction and position microbial indole metabolites as key mediators of diet–microbiota–host crosstalk in intestinal homeostasis.

## Data Availability

The 16S rRNA gene sequencing data have been deposited into the NCBI Sequence Read Archive (SRA) database with the accession numbers PRJNA1493071 (https://www.ncbi.nlm.nih.gov/sra/PRJNA1493071, URA (accessed on 1 January 2026)) Untargeted Metabolomics data are available in Metabolomics Workbench (http://dx.doi.org/10.21228/M88K3C, URA (accessed on 1 January 2026)). Single-cell RNA sequencing data are available in the NCBI SRA database with the accession numbers PRJNA1495254 (https://www.ncbi.nlm.nih.gov/sra/PRJNA1495254, URA (accessed on 1 January 2026)).

## Supplementary Materials

The following supporting information can be downloaded at https://www.mdpi.com/article/10.3390/microorganisms14102280/s1, Figure S1: The potential role of GLSP-diet in the Intestine of mice; Figure S2: The intestinal protective effect of GLSP-diet on 8-month-old mice; Figure S3: GLSP-diet alleviates DSS-induced acute intestinal injury in mice; Figure S4: GLSP-diet reshaped the gut microbiota of mice; Figure S5: Quality assessment of non-target metabolomics data.

## Abbreviations

The following abbreviations are used in this manuscript:

ABX	Antibiotics
DAI	Disease activity index
DSS	Dextran sulfate sodium
FMT	Fecal microbiota transplantation
GLSP	*Ganoderma lucidum* sporoderm-broken spore powder
ISCs	Intestinal stem cells
SCFAs	Short-chain fatty acids
PCA	Principal component analysis
TA	Transit-amplifying (cells)
UPGMA	Unweighted pair group method with arithmetic mean
