# Supplementary material for: Ganoderma lucidum Sporoderm-Broken Spore Powder (GLSP) Remodels the Gut Microbiota and Promotes Tryptophan-Derived Indole Metabolites to Maintain Intestinal Homeostasis
Source: Microorganisms. 2026 Oct 7;14(10):2280. doi: 10.3390/microorganisms14102280 (PMC13707510; doi:10.3390/microorganisms14102280)
Supplement: Supplementary file 1 [file microorganisms-14-02280-s001.zip › microorganisms-4546524-supplementary.pdf]

Supplementary Figures

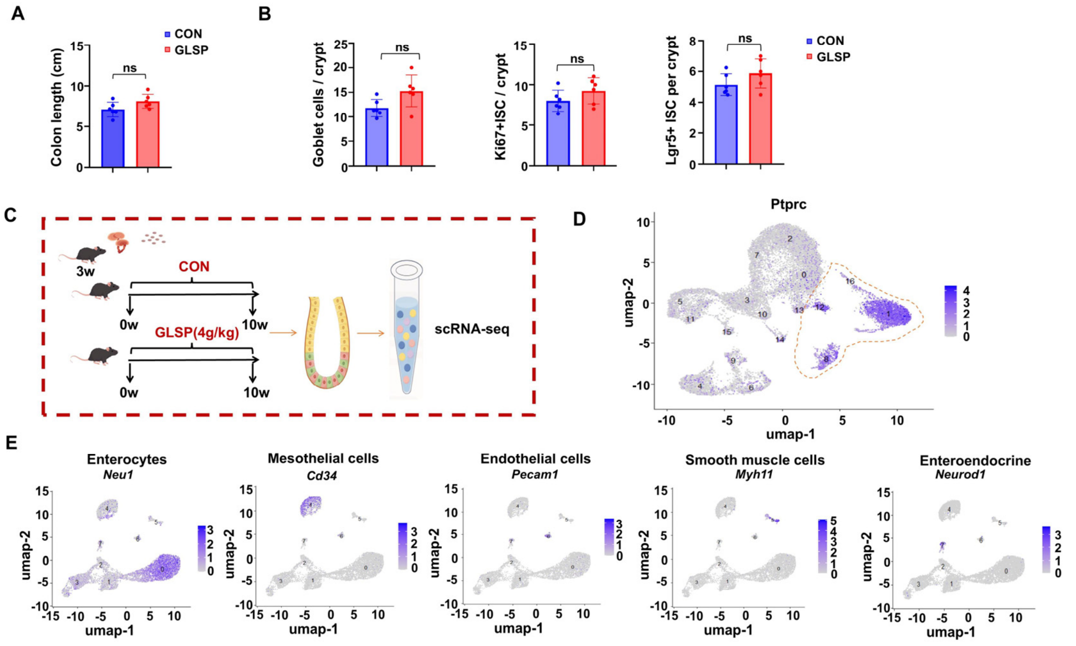

Figure S1: The potential role of GLSP-diet in the Intestine of mice.

(A) Statistical analysis of colon length (n = 6). **(B)** Number of goblet cells, Ki-67-positive cells, and Lgr5-positive cells expression (n = 6). **(C)** Schematic diagram of the animal experiment. **(D)** UMAP plot of CD45 distinguishing epithelial and immune cells. **(E)** UMAP plots showing the expression levels of signature genes in different clusters.

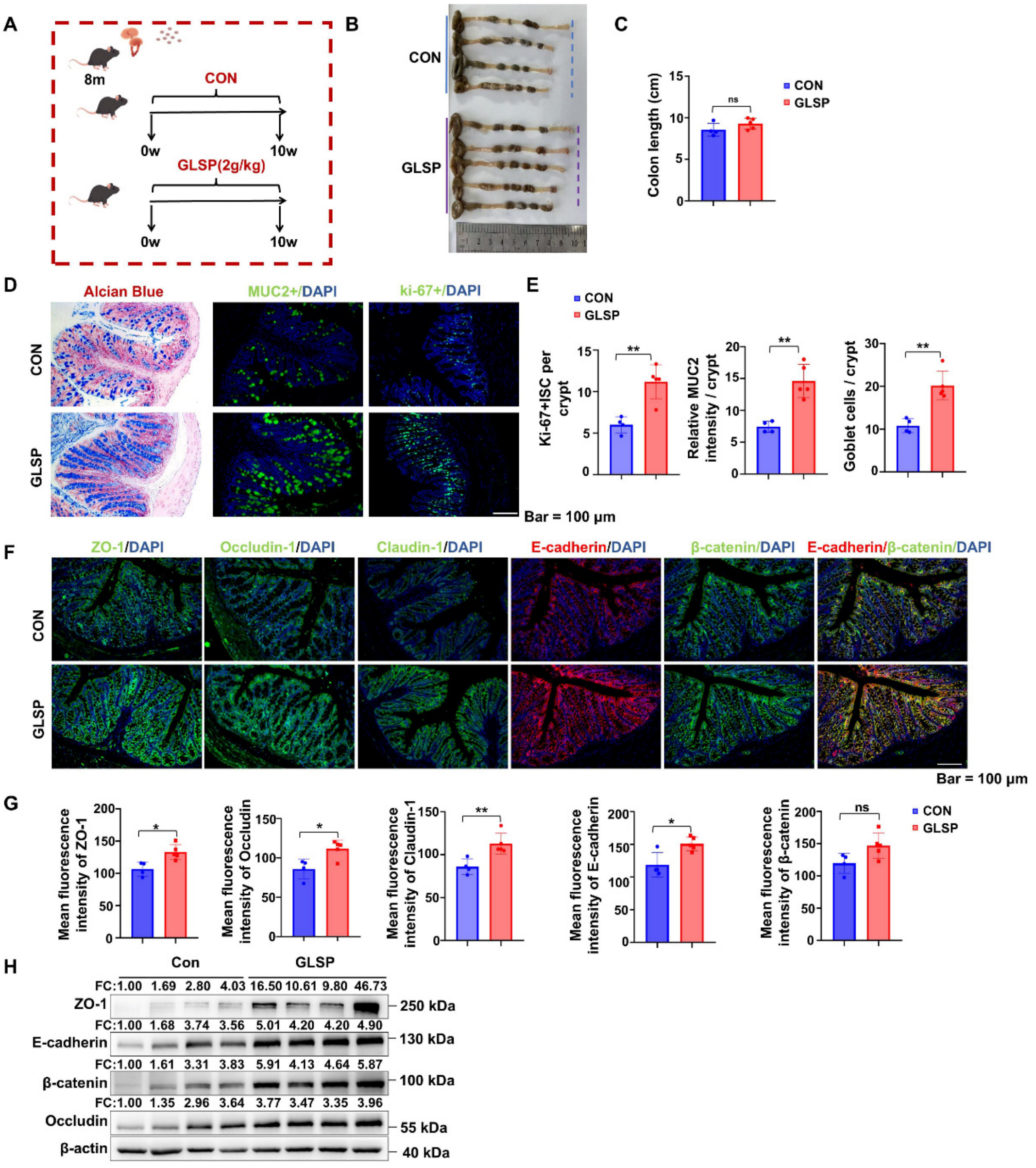

Figure S2. The intestinal protective effect of GLSP-diet on 8-month-old mice.

(A) Schematic diagram of the animal experiment. **(B, C)** Representative images of colonic tissues and statistical analysis of colonic length (n = 4/5). **(D)** Colonic sections stained with Alcian Blue, alongside immunofluorescent staining for MUC2 and Ki-67 (n = 4/5). **(E)** Number of goblet cells and relative expression levels of MUC2 Ki-67 (n = 4/5). **(F, G)** Representative immunofluorescence images and mean fluorescence intensity of ZO-1, Occludin, Claudin-1, E-cadherin, and β-catenin in colonic sections (n = 4/5). **(H)** Western blot analysis of ZO-1, E-cadherin, β-catenin and occluding in in colonic tissues. The band intensities were normalized to β-actin, and the relative expression (Fold change, FC) refers to the ratio relative to the control group. ns, non-significant; *p < 0.05, **p < 0.01. Scale bar, 100μm.

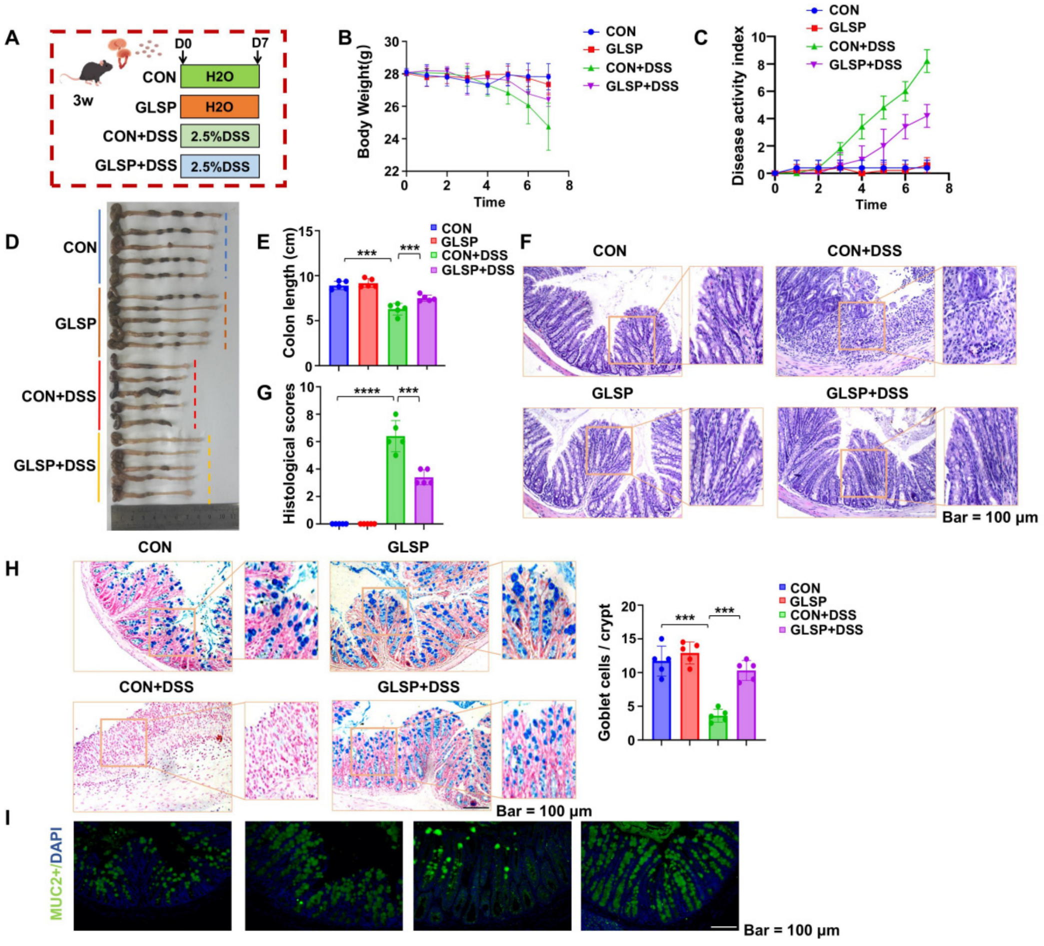

Figure S3. GLSP-diet alleviates DSS-induced acute intestinal injury in mice.

(A) Schematic diagram of the animal experiment. **(B)** Body weight change of mice (n = 5). **(C)** DAI score (n = 5). **(D, E)** Representative images and length of colonic tissues (n = 5). **(F, G)** Representative images and histological scores colonic sections stained with H&E (n = 5). **(H, I)** Colon sections stained with Alcian Blue and immunofluorescent MUC2 (n = 5). ***$p < 0.001$, ****$p < 0.0001$. Scale bar, 100μm.

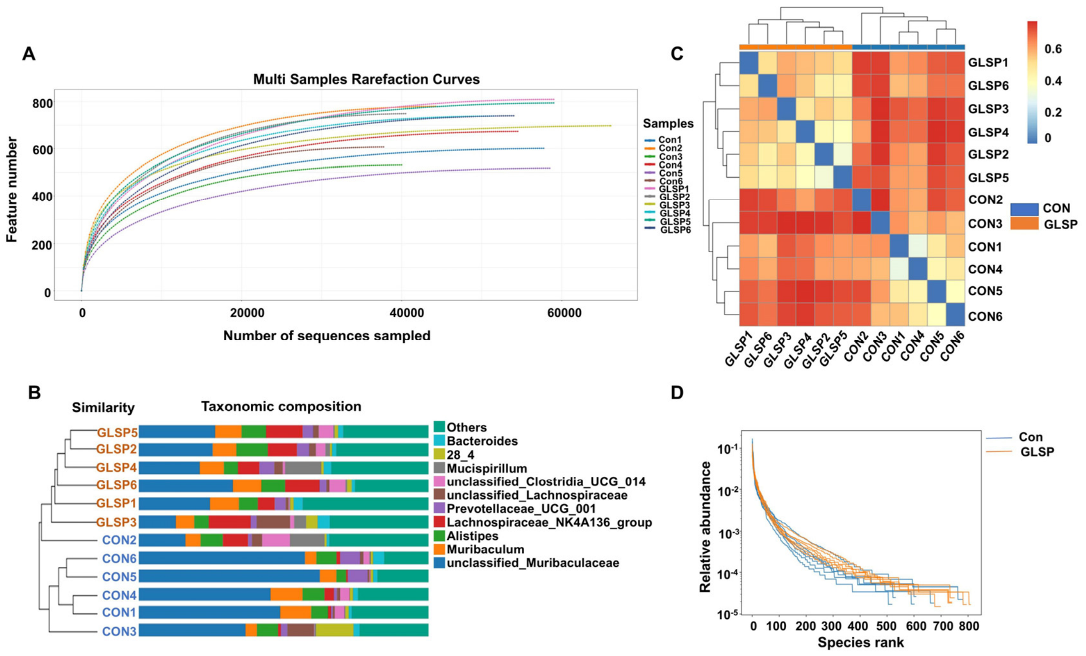

Figure S4. GLSP-diet reshaped the gut microbiota of mice.

(A) Rarefaction Curve (n = 6). **(B)** UPGMA clustering tree and column chart of relative species abundance (n = 6). **(C)** Sample clustering heat map (n = 6). **(D)** Rank Abundance Curve (n = 6).

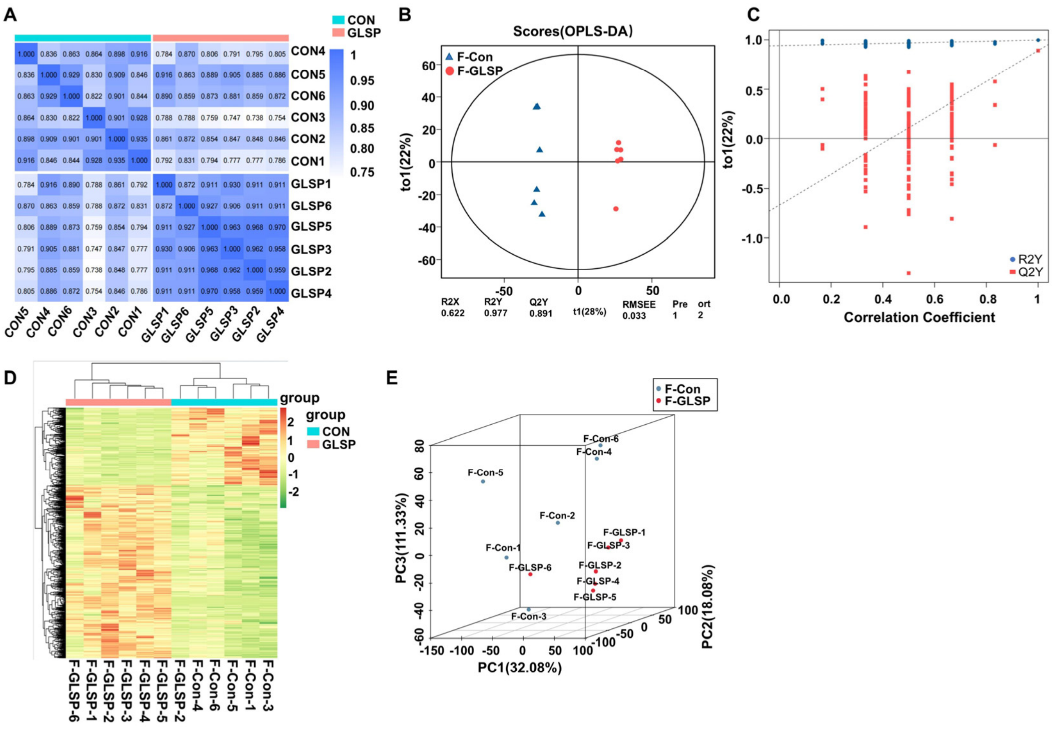

Figure S5: Quality assessment of non-target metabolomics data

(A) Correlation plot between samples (n = 6). **(B, C)** OPLS-DA Analysis Plot (n = 6). **(D)** Hierarchical clustering analysis heatmap of differential metabolites (n = 6). **(E)** Principal Component Analysis (PCA) Score Plot (n = 6).
